# *In Vitro* and *In Vivo* Efficacy of Ether Lipid Edelfosine against *Leishmania spp.* and SbV-Resistant Parasites

**DOI:** 10.1371/journal.pntd.0001612

**Published:** 2012-04-10

**Authors:** Rubén E. Varela-M, Janny A. Villa-Pulgarin, Edward Yepes, Ingrid Müller, Manuel Modolell, Diana L. Muñoz, Sara M. Robledo, Carlos E. Muskus, Julio López-Abán, Antonio Muro, Iván D. Vélez, Faustino Mollinedo

**Affiliations:** 1 Instituto de Biología Molecular y Celular del Cáncer, Centro de Investigación del Cáncer, CSIC-Universidad de Salamanca, Campus Miguel de Unamuno, Salamanca, Spain; 2 APOINTECH, Centro Hispano-Luso de Investigaciones Agrarias, Parque Científico de la Universidad de Salamanca, Villamayor, Salamanca, Spain; 3 Laboratorio de Inmunología Parasitaria y Molecular, CIETUS, Facultad de Farmacia, Universidad de Salamanca, Campus Miguel de Unamuno, Salamanca, Spain; 4 Department of Medicine, Section of Immunology, St. Mary's Campus, Imperial College London, London, United Kingdom; 5 Department of Cellular Immunology, Max-Planck-Institut für Immunbiologie, Freiburg, Germany; 6 Programa de Estudio y Control de Enfermedades Tropicales, Universidad de Antioquia, Medellín, Colombia; New York University School of Medicine, United States of America

## Abstract

**Background:**

The leishmaniases are a complex of neglected tropical diseases caused by more than 20 *Leishmania* parasite species, for which available therapeutic arsenal is scarce and unsatisfactory. Pentavalent antimonials (SbV) are currently the first-line pharmacologic therapy for leishmaniasis worldwide, but resistance to these compounds is increasingly reported. Alkyl-lysophospoholipid analogs (ALPs) constitute a family of compounds with antileishmanial activity, and one of its members, miltefosine, has been approved as the first oral treatment for visceral and cutaneous leishmaniasis. However, its clinical use can be challenged by less impressive efficiency in patients infected with some *Leishmania* species, including *L. braziliensis* and *L. mexicana*, and by proneness to develop drug resistance *in vitro*.

**Methodology/Principal Findings:**

We found that ALPs ranked edelfosine>perifosine>miltefosine>erucylphosphocholine for their antileishmanial activity and capacity to promote apoptosis-like parasitic cell death in promastigote and amastigote forms of distinct *Leishmania spp.*, as assessed by proliferation and flow cytometry assays. Effective antileishmanial ALP concentrations were dependent on both the parasite species and their development stage. Edelfosine accumulated in and killed intracellular *Leishmania* parasites within macrophages. *In vivo* antileishmanial activity was demonstrated following oral treatment with edelfosine of mice and hamsters infected with *L. major*, *L. panamensis* or *L. braziliensis*, without any significant side-effect. Edelfosine also killed SbV-resistant *Leishmania* parasites in *in vitro* and *in vivo* assays, and required longer incubation times than miltefosine to generate drug resistance.

**Conclusions/Significance:**

Our data reveal that edelfosine is the most potent ALP in killing different *Leishmania spp.*, and it is less prone to lead to drug resistance development than miltefosine. Edelfosine is effective in killing *Leishmania* in culture and within macrophages, as well as in animal models infected with different *Leishmania spp.* and SbV-resistant parasites. Our results indicate that edelfosine is a promising orally administered antileishmanial drug for clinical evaluation.

## Author Summary

Leishmaniasis represents a major international health problem, has a high morbidity and mortality rate, and is classified as an emerging and uncontrolled disease by the World Health Organization. The migration of population from endemic to nonendemic areas, and tourist activities in endemic regions are spreading the disease to new areas. Unfortunately, treatment of leishmaniasis is far from satisfactory, with only a few drugs available that show significant side-effects. Here, we show *in vitro* and *in vivo* evidence for the antileishmanial activity of the ether phospholipid edelfosine, being effective against a wide number of *Leishmania spp.* causing cutaneous, mucocutaneous and visceral leishmaniasis. Our experimental mouse and hamster models demonstrated not only a significant antileishmanial activity of edelfosine oral administration against different wild-type *Leishmania spp.*, but also against parasites resistant to pentavalent antimonials, which constitute the first line of treatment worldwide. In addition, edelfosine exerted a higher antileishmanial activity and a lower proneness to generate drug resistance than miltefosine, the first drug against leishmaniasis that can be administered orally. These data, together with our previous findings, showing an anti-inflammatory action and a very low toxicity profile, suggest that edelfosine is a promising orally administered drug for leishmaniasis, thus warranting clinical evaluation.

## Introduction

The impact of the leishmaniases on human health has been grossly underestimated for many years, and this complex of diseases has been classified by the World Health Organization (WHO) as one of the most neglected tropical diseases [1]. During the last decade, endemic areas have been spreading and a sharp increase in the number of leishmaniasis cases has been recorded. The WHO classifies leishmaniasis as a category 1 disease (“emerging and uncontrolled”), and there is an urgent need to develop new therapeutic drugs and approaches. Currently, about 350 million people in 98 countries around the world are at risk, and an estimated 12 million people are infected [1]. Despite progress in the diagnosis and treatment, leishmaniasis remains a major public health problem, particularly in tropical and sub-tropical developing countries. Published figures indicate an estimated incidence of two million new cases per year, with 1.5 million cases of self-healing, but disfiguring, cutaneous leishmaniasis, and 500,000 cases of life-threatening visceral leishmaniasis [1], [2]. Approximately 60,000 people die from visceral leishmaniasis each year, a rate surpassed among parasitic diseases only by malaria; and a loss of about 2.4 million disability-adjusted life years (DALYs) throughout the world has been calculated as the total disease burden of leishmaniasis [1]–[3]. Furthermore, a number of reports have emphasized the increasing importance of visceral leishmaniasis as an opportunistic infection among HIV-positive patients in areas where both infections are endemic [4].

The chemotherapy currently available for the leishmaniases is far from satisfactory and presents several problems, including toxicity, many adverse side-effects, high costs and development of drug resistance [2], [5]. Two pentavalent antimonial (SbV) compounds, sodium stibogluconate (Pentostam) and meglumine antimoniate (Glucantime), were first introduced in the 1940's and have since been used as first-line chemotherapeutic agents against all forms of leishmaniasis through parenteral administration. Although SbV, administered by intramuscular or intravenous route, remains the first-line drug for the treatment of leishmaniasis worldwide, its efficacy is becoming increasingly lower [6], and highly depends on *Leishmania* species and distinct endemic regional variations, even within the same country. Resistance is now common in India, and rates of resistance have been shown to be higher than 60% in parts of the state of Bihar, in north-east India [7], [8]. In addition, the incidence of adverse effects, including myalgia, arthralgias, pancreatitis, nephrotoxicity, hepatotoxicity, and cardiotoxicity [1], [2], [9], makes the search for new alternative medicines to SbV an urgent issue, and a number of drugs are now in clinical trials [10]. Intravenous infusion of liposomal amphotericin B (AmBisome) is at present the most effective anti-*Leishmania* drug [2], [11], but its relatively high cost makes it unaffordable in several poor areas of the world where the disease is more prevalent [2]. In addition, the requirement for long periods of parenteral administration, frequently requiring hospitalization, has also limited the clinical use of amphotericin B.

Miltefosine (Impavido) is a new oral agent that has shown high cure rates in visceral leishmaniasis in India (*L. donovani*; 94% cure) [12], and in cutaneous leishmaniasis in Colombia (*L. panamensis*; >90% cure) [13]. However, a recent therapeutical trial has revealed a limited potential of miltefosine for the treatment of American cutaneous leishmaniasis, with an unsatisfactory cure rate of 69.8% in Colombia [14]. Furthermore, this percentage fell to 49% when miltefosine was administered to patients with lesions caused by *L. braziliensis*, which comprise more than 60% of cutaneous leishmaniasis in Colombia [14]. Additional recent clinical trials in Brazil showed a cure rate of miltefosine for the treatment of cutaneous leishmaniasis caused by *L. braziliensis* of 75% [15], and for the treatment of cutaneous leishmaniasis caused by *L. guyanensis* of 71% [16]. Miltefosine treatment also led to approximately 70% cure rate for mucosal leishmaniasis due to *L. braziliensis* in Bolivia [17], [18]. Moreover, the miltefosine cure rate was approximately 53% for cutaneous leishmaniasis (33% for *L. braziliensis* infection, and 60% for *L. mexicana* infection) in Guatemala [13], [19], [20], and a cure rate of 63% was reported for *L. tropica* in Afghanistan [20]. These figures contrast with cure rates of more than 82% in the treatment of visceral leishmaniasis (kala-azar) in India [21], [22] and Bangladesh [23]. These data point out the great variability in the outcome depending on the geographical area for reasons that are not well understood. In addition, miltefosine commonly induces gastrointestinal side-effects, such as anorexia, nausea, vomiting and diarrhea, that sometimes lead to drop out from treatment [1], [2], [22]. Miltefosine is potentially teratogenic and should not be administered to pregnant women [1], [2], for whom adequate contraception should be guaranteed during treatment and for up to 3 months afterwards [1], given the teratogenic potential of miltefosine in animal models [24]. An additional concern is the rapid *in vitro* generation of resistance to miltefosine [25]–[27] that could limit its clinical use. Thus, these studies reinforce the need to search for new therapeutic alternatives in the treatment of leishmaniasis.

Edelfosine (1-*O*-octadecyl-2-*O*-methyl-*rac*-glycero-3-phosphocholine, ET-18-OCH_3_) is a promising antitumor ether lipid drug [28]–[30], which is not mutagenic and acts by activating apoptosis through its interaction with cell membranes [31]–[34]. In addition to its antitumor activity, edelfosine has been shown to exert *in vitro* antiparasitic activity against different species of *Leishmania* parasites [35]–[37]. Edelfosine has been considered the prototype molecule of a rather heterogeneous family of synthetic compounds collectively known as alkyl-lysophospholipid analogs (ALPs), that comprise the above clinically relevant miltefosine as well as perifosine, which also shows anti-*Leishmania* activity [38], [39]. Although the mechanism of action of miltefosine against *Leishmania* parasites remains to be fully elucidated, there are some reports showing that the ability of this compound to promote an apoptosis-like cell death is critical for its leishmanicidal activity [40], [41]. Because edelfosine has been shown to have a higher proapototic activity than both miltefosine and perifosine in human cancer cells [29], [30], [33], we have carried out here a comprehensive *in vitro* and *in vivo* study, investigating the putative anti-*Leishmania* traits of edelfosine, as compared to other ALPs, using different *Leishmania* species as well as mouse and hamster experimental models.

## Materials and Methods

### Ethics statement

Animal procedures in this study complied with the Spanish (Real Decreto RD1201/05) and the European Union (European Directive 2010/63/EU) guidelines on animal experimentation for the protection and humane use of laboratory animals, and were conducted at the accredited Animal Experimentation Facility (Servicio de Experimentación Animal) of the University of Salamanca (Register number: PAE/SA/001). Procedures were approved by the Ethics Committee of the University of Salamanca (protocol approval number 48531).

### Drugs

Edelfosine (1-*O*-octadecyl-2-*O*-methyl-*rac*-glycero-3-phosphocholine) was from INKEYSA (Barcelona, Spain) and Apointech (Salamanca, Spain). Miltefosine (hexadecylphosphocholine) was from Calbiochem (Cambridge, MA). Perifosine (octadecyl-(1,1-dimethyl-piperidinio-4-yl)-phosphate) and erucylphosphocholine ((13Z)-docos-13-en-1-yl 2-(trimethylammonio)ethyl phosphate) were from Zentaris (Frankfurt, Germany). Stock sterile solutions of the distinct ALPs (2 mM) were prepared in RPMI-1640 culture medium (Invitrogen, Carlsbad, CA), supplemented with 10% (v/v) heat-inactivated fetal bovine serum (FBS), 2 mM L-glutamine, 100 units/ml penicillin, and 100 µg/ml streptomycin (GIBCO-BRL, Gaithersburg, MD) as previously described [28].

### *Leishmania* cells and culture conditions

The following *Leishmania* strains were used in this study: *L. amazonensis* (MHOM/Br/73/LV78), *L. braziliensis* (MHOM/CO/88/UA301), *L. donovani* (MHOM/IN/80/DD8), *L. infantum* (MCAN/ES/96/BCN150), *L. major* LV39 (MRHO/SU/59/P), *L. mexicana* (MHOM/MX/95/NAN1), and *L. panamensis* (MHOM/CO/87/UA140).

*Leishmania* promastigotes were grown in RPMI-1640 culture medium, supplemented with 10% FBS, 2 mM glutamine, 100 units/ml penicillin, and 100 µg/ml streptomycin at 26°C. Promastigotes were treated with the indicated compounds during their logarithmic growth phase (1.5×10^6^ parasites/ml) at 26°C. Late stationary promastigotes were obtained after incubation of the parasites for 5–6 days with starting inocula of 1×10^6^ parasites/ml. *Leishmania* axenic amastigotes were obtained at pH 5.0 in Schneider's culture medium following a stepped temperature increase to 30, 31 and 32°C, except for *L. infantum* amastigotes, which were exposed to 34, 36 and 37°C, as previously described [42].

### Growth inhibition assay

The antileishmanial activity in promastigotes and axenic amastigotes was determined by using the XTT (sodium 3,3′-[1-(phenylaminocarbonyl)-3,4-tetrazolium]-bis (4-methoxy-6-nitro) benzene sulfonic acid hydrate) cell proliferation kit (Roche Molecular Biochemicals, Mannheim, Germany) as previously described [42], [43]. Cells were resuspended in FBS-containing RPMI-1640 culture medium (1.5×10^6^ cells/ml for promastigotes, and 2×10^6^ cells/ml for axenic amastigotes), and plated (100 µl/well) in 96-well flat-bottomed microtiter plates at 26°C, in the absence and in the presence of different concentrations of the indicated ALPs. After 72-h incubation at 26°C, IC_50_ (half-maximal inhibitory concentration) values, defined as the drug concentration causing 50% inhibition in cell proliferation with respect to untreated controls, were determined for each compound. Measurements were done in triplicate, and each experiment was repeated four times.

### Analysis of apoptosis-like cell death by flow cytometry

One and a half million *Leishmania spp.* promastigotes or axenic amastigotes were treated in the absence and in the presence of the indicated concentrations of ALPs for different incubation times. Then, parasites were pelleted by centrifugation (1000× *g*) for 5 min, and analyzed for apoptosis-like DNA breakdown by flow cytometry following a protocol previously described [44]. Quantitation of apoptotic-like cells was monitored as the percentage of cells in the sub-G_0_/G_1_ region (hypodiploidy) in cell cycle analysis [44], [45], using a fluorescence-activated cell sorting (FACS) Calibur flow cytometer (Becton Dickinson, San Jose, CA) equipped with a 488 nm argon laser. WinMDI 2.8 software was used for data analysis.

### Intracellular distribution of fluorescent edelfosine analog in *L. panamensis*–infected J774 macrophages

The mouse macrophage-like cell line J774, grown in RPMI-1640 culture medium, supplemented with 10% FBS, 2 mM L-glutamine, 100 U/mL penicillin, and 100 µg/ml streptomycin, at 37°C in humidified 95% air and 5% CO_2_, was infected overnight at the exponential growth phase (3×10^5^ cells/ml) with stationary-phase *L. panamensis* promastigotes, at a macropage/promastigote ratio of 1/10 in complete RPMI-1640 culture medium. Non-internalized promastigotes were removed by 2–3 successive washes with PBS. Then, uninfected and *L. panamensis*-infected J774 macrophages were incubated for 1 h with 10 µM of the fluorescent edelfosine analog all-(E)-1-*O*-(15′-phenylpentadeca-8′,10′,12′,14′-tetraenyl)-2-*O*-methyl-*rac*-glycero-3-phosphocholine (PTE-ET) [34], [46], [47] (kindly provided by F. Amat-Guerri and A.U. Acuña, Consejo Superior de Investigaciones Científicas, Madrid, Spain) in complete RPMI-1640 culture medium. In addition, J774 cells were also incubated first with 10 µM PTE-ET for 1 h, then washed with PBS and infected with *L. panamensis* in the darkness for 6 h. Samples were fixed with 1% formaldehyde, and analyzed with a Zeiss Axioplan 2 fluorescence microscope (Carl Zeiss GmbH, Oberkochen, Germany) (40× magnification).

### Assessment of intracellular parasitic load in macrophage-like cells

J774 cells were infected with *L. panamensis* promastigotes as above. The number of intracellular viable parasites was assessed by incubating infected cells with RPMI-1640 medium containing 0.008% SDS to gently disrupt macrophage plasma membrane, followed by addition of RPMI-1640 culture medium containing 20% FBS to stop further lysis. Samples were then sequentially diluted in 96-well plates containing biphasic Novy-MacNeal-Nicolle (NNN) medium. Plates were incubated at 26°C for 20 days, and examined weekly under an inverted Nikon TS-100 microscope (Nikon, Kanagawa, Japan) to evaluate the presence of viable motile promastigotes. The reciprocal of the highest dilution found positive for parasite growth was considered to be the concentration of parasites.

### Determination of nitric oxide (NO) by the nitrite assay

Macrophage-like J774 cells were plated in complete RPMI-1640 culture medium at a concentration of 1×10^6^ cells/well in 24-well culture plates (Costar, Cambridge, MA), and let them adhere for 2 h at 37°C in 5% CO_2_. Non-adhering cells were removed by gentle washing with complete RPMI-1640 culture medium. Adherent J774 cells were incubated in the absence (negative control), or in the presence of 10 µg/ml lipopolysaccharide (Sigma, St. Louis, MO) (LPS; positive control) or of different concentrations of edelfosine. After 18-h incubation at 37°C in 5% CO_2_, supernatants were collected, centrifuged at 500× *g* for 10 min, and stored at −80°C until analysis. NO release was indirectly measured using a colorimetric assay based on the Griess reaction. Triplicate 100-µl aliquots of cell culture supernatants were incubated with 50 µl of freshly prepared Griess reagent (1% sulfanilamide, 0.1% naphthylethylene diamide dihydrochloride, and 2.5% orthophosphoric acid) for 15 min at room temperature, and then absorbance of the azo-chromophore was measured at 550 nm. Nitrite concentration was determined by using sodium nitrite as a standard. All samples were assayed against a blank comprising complete RPMI-1640 culture medium incubated for 18 h on the same plates as the samples, but in the absence of cells. All reagents were purchased from Sigma. Results were expressed in nanomoles of nitrite per 10^6^ macrophages.

### Evaluation of antileishmanial activity in mouse and hamster models

Six-week-old female BALB/c mice (18–20 g) and four-week-old male Syrian golden hamsters (*Mesocricetus auratus*) (about 120 g) (Charles River Laboratories, Lyon, France), kept in a pathogen-free facility and handled according to institutional guidelines, complying with the Spanish legislation under a 12/12-h light/dark cycle at a temperature of 22°C, received a standard diet and water *ad libitum*. Mice were inoculated *s.c.* into their left hind footpad (in a total volume of 50 µl PBS) with 2×10^6^ infective stationary-phase promastigotes, whereas hamsters, previously anesthetized with inhaled Forane, were inoculated intradermally in the nose with 1×10^6^ stationary-phase promastigotes in a volume of 50 µl PBS. When inflamation was evident (about 1 week in mice, and 6 weeks in hamsters, after inoculation), animals were randomly assigned into cohorts of 7 animals each, receiving a daily oral administration (through a feeding needle) of edelfosine (15 mg/kg for mice, and 26 mg/kg for hamsters, in water), or an equal volume of vehicle (water). In mice, the footpad thickness was measured with calipers every week, and compared with the uninfected right hind footpad to obtain the net increase in footpad swelling. In hamsters, nose swelling was measured with calipers every week, and compared with the nose size before inoculation and treatment. Evolution index of the lesion was calculated as size of the lesion during treatment (mm)/size of the lesion before treatment. Animal body weight and any sign of morbidity were monitored. Drug treatment lasted for 28 days, and animals were killed following institutional guidelines, 24 h after the last drug administration.

After the killing of the animals, the parasite burden in the infected tissues was determined by limiting dilution assays as previously described [48]. Biopsies were washed 3 times with PBS supplemented with 100 units/ml penicillin and 100 µg/ml streptomycin (GIBCO-BRL), and then incubated overnight (12 h) at 4°C with PBS containing 100 units/ml of penicillin and 100 µg/ml streptomycin. Following overnight incubation, biopsies were washed 2–3 times with PBS supplemented with the above antibiotics, and then a weighed piece of the infected area was homogenized in 1 ml PBS containing antibiotics using a sterile glass Potter-Elvejhem type tissue grinder. Homogenate was diluted at a final concentration of 0.1 mg/ml in Schneider's culture medium, containing 100 units/ml penicillin and 100 µg/ml streptomycin; and then serial dilutions were made in triplicate in 96-well plates containing biphasic Novy-MacNeal-Nicolle (NNN) medium. Plates were incubated at 26°C for 20 days, and examined weekly under an inverted Nikon TS-100 microscope to evaluate the presence of viable promastigotes. The reciprocal of the highest dilution found positive for parasite growth was considered to be the concentration of parasites per mg of tissue. Total parasite load was calculated using the total weight of the respective infected organ.

### Induction of *in vitro* resistance to Glucantime in *L. panamensis* promastigotes

Parasites cultured in Schneider's culture medium supplemented with 10% FBS, 100 units/ml penicillin, and 100 µg/ml streptomycin at 26°C for 5 days, were washed twice with PBS, and centrifuged at 1000× *g* for 10 min at room temperature. Parasites were then resuspended at 2×10^6^ promastigotes/ml in Schneider's culture medium, and incubated at 26°C for 5 days with 4 mg/ml Glucantime (Aventis Pharma, Sao Paulo, Brazil), which corresponded to its IC_50_ value, previously assessed by the XTT technique. Drug-containing culture medium was changed every 4–6 days, depending on parasite growth, and parasites were washed with PBS, analyzed by XTT assay, and resuspended again at 2×10^6^ parasites/ml. This procedure was repeated until parasite viability in the presence of the drug was over 80%. Then, after achieving this viability rate, this process was repeated three times, with increasing concentrations of SbV, up to reaching a final concentration of 37 mg/ml. The volume of drug solution used in each passage was controlled not to exceed 10% of the total volume of culture medium.

### Assessment of *L. panamensis* resistance to SbV in the hamster animal model

The level of SbV resistance was further assessed by infection of golden hamsters with the above *in vitro*-generated SbV-resistant (SbV-R) *L. panamensis* parasites, growing in the presence of 37 mg/ml SbV, as well as with wild-type susceptible *L. panamensis*, followed by treatment with Glucantime. Hamsters were divided into two groups, eight animals infected with the resistant strain and eight animals infected with the susceptible strain. Each group was inoculated intradermally on the nose with 1×10^6^ stationary-phase promastigotes in a volume of 50 µl PBS. These animals were previously anesthetized with ketamine (50 mg/ml) and xylazine (5 mg/kg) intraperitoneally. About six weeks after infection, lesions were evident in both animal groups, and animals were treated daily with 40 mg/kg Glucantime, intramuscularly using a 27-gauge needle, for ten days. Evolution of the lesions and drug efficacy were monitored as above.

### Induction of *in vitro* resistance to ALPs in different *Leishmania* species

ALP-resistant *Leishmania* strains were generated as indicated above for SbV-resistant parasites. Drugs were initially incubated at their corresponding IC_50_ values, and then drug concentration was gradually increased. Parasites were considered resistant when they could grow at a drug concentration of 30 µM.

### Statistical analysis

Data are shown as mean ± SD. Between-group statistical differences were assessed using the Mann-Whitney or the Student's *t* test. A *P*-value of <0.05 was considered statistically significant.

## Results

### ALPs differentially inhibit the proliferation of *Leishmania spp.* promastigotes

**Figure 1:**
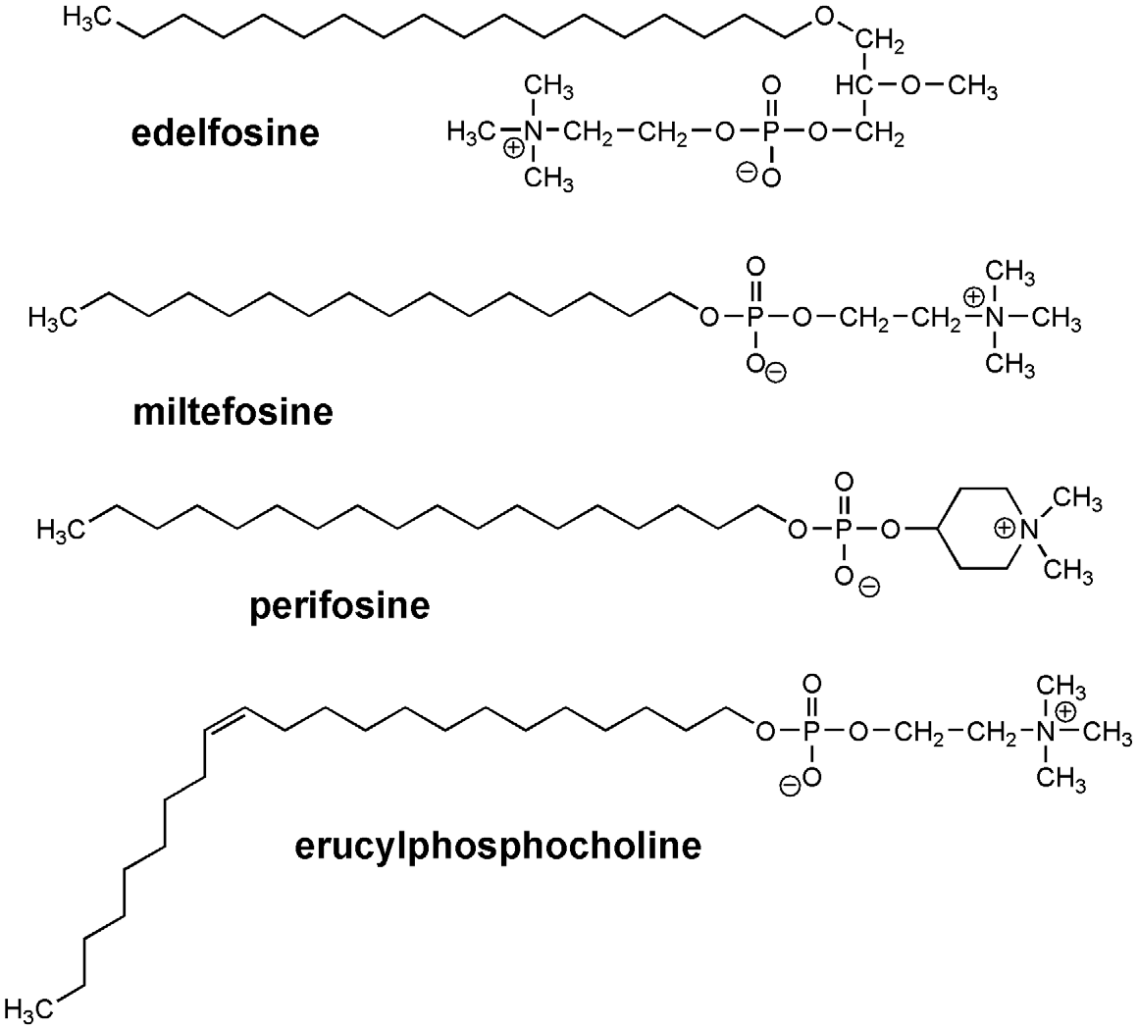
Chemical structures of edelfosine, miltefosine, perifosine and erucylphosphocholine.

We analyzed the antileishmanial potential of the four most clinically relevant ALPs, namely edelfosine, miltefosine, perifosine and erucylphosphocholine (Figure 1). By using the XTT assay, we found that edelfosine and perifosine were the most active ALPs inhibiting proliferation of distinct *Leishmania spp.* promastigotes with IC_50_ values in the range of low micromolar concentration (∼2–9 µM) in most cases (*L. donovani*, *L. panamensis*, *L. mexicana*, *L. major*, *L. amazonensis*) (Table 1). *L. braziliensis* and *L. infantum* promastigotes were more resistant to the action of edelfosine, perifosine and miltefosine than the other *Leishmania* species tested (Table 1). Erucylphosphocholine was the least efficient ALP in inhibiting parasite proliferation regarding most *Leishmania spp.* promastigotes, but interestingly it showed the highest antiparasitic activity against *L. infantum* promastigotes (Table 1). In general, the antileishmanial activity of the distinct ALPs ranked edelfosine≥perifosine>miltefosine>erucylphosphocholine against *Leishmania spp.* promastigotes.

**Table 1:** Inhibition of proliferation of different *Leishmania spp.* (IC_50_ values) by ALPs.

Parasite stage	IC_50_ (µM)			
Promastigotes	Edelfosine	Miltefosine	Perifosine	ErPC
*L. amazonensis*	6.4±0.3	13.0±0.8	9.6±1.8	40.0±3.0
*L. braziliensis*	18.3±3.7	37.7±3.2	14.3±2.1	21.0±3.5
*L. donovani*	2.1±0.3	3.1±0.8	2.2±0.4	13.3±1.2
*L. infantum*	27.7±4.6	47.3±4.1	35.3±2.5	16.7±2.8
*L. major*	2.0±0.2	6.8±0.3	7.1±0.5	12.7±1.5
*L. mexicana*	2.4±0.2	2.7±0.7	2.5±0.1	11.1±4.9
*L. panamensis*	2.3±0.8	6.3±0.6	2.4±0.2	14.1±3.1

	IC_50_ (µM)			
Axenic amastigotes	Edelfosine	Miltefosine	Perifosine	ErPC
*L. amazonensis*	3.1±0.1	5.9±1.2	2.9±0.5	42.9±3.9
*L. braziliensis*	8.1±0.2	6.5±1.2	15.1±3.0	28.2±0.7
*L. donovani*	5.3±0.2	14.5±0.5	9.9±0.7	47.7±3.9
*L. infantum*	4.2±0.6	4.4±1.1	1.7±0.2	37.0±3.1
*L. mexicana*	5.2±1.4	4.4±0.8	2.5±0.8	37.6±7.8
*L. panamensis*	12.2±1.2	39.3±3.7	9.3±0.8	65.8±6.7

*Leishmania* parasites were incubated with edelfosine, miltefosine, perifosine and erucylphosphocholine (ErPC), and assayed for growth inhibition by XTT assays as described in Materials and Methods. Data are shown as the mean values ± SD of four independent determinations.

### Effect of ALPs in inhibiting proliferation of *Leishmania* spp. axenic amastigotes

Next, we analyzed the antileishmanial activity of the distinct ALPs against distinct axenic *Leishmania* amastigotes. Following an axenic amastigote drug screening, we found that edelfosine and perifosine behaved as the most potent ALPs in the inhibition of proliferation of distinct *Leishmania spp.* amastigotes (Table 1). A wider range of IC_50_ values was detected for amastigote than for promastigote forms of *Leishmania* (Table 1). The IC_50_ values for the anti-*Leishmania* amastigote activity of edelfosine and perifosine ranged between ∼3–12 µM and ∼2–15 µM, respectively. Miltefosine showed a higher degree of variability (IC_50_, ∼4–39 µM), with *L. panamensis* amastigotes being rather resistant (IC_50_, 39.3 µM) (Table 1). Erucylphosphocholine showed the highest IC_50_ values (∼28–66 µM) for the inhibition of cell growth in all the *Leishmania spp.* amastigotes analyzed (Table 1). Surprisingly, *L. infantum* amastigotes were very sensitive to the action of perifosine, edelfosine and miltefosine, whereas their cognate promastigotes forms were rather resistant (Table 1), with double digit IC_50_ figures for promastigotes and low one-digit IC_50_ values for amastigotes. Interestingly, *L. braziliensis* amastigotes were far more sensitive to edelfosine and miltefosine than their promastigote counterparts (Table 1), whereas perifosine and erucylphosphocholine showed similar IC_50_ values for both *L. braziliensis* promastigote and amastigote forms with IC_50_ figures over 14 µM (Table 1). In general, the antileishmanial activity of the distinct ALPs ranked edelfosine≥perifosine>miltefosine>erucylphosphocholine against *Leishmania spp.* amastigotes. These results indicate that sensitivity of *Leishmania* parasites to ALPs is highly dependent on each species as well as on their stage form, namely promastigote or amastigote. Interestingly, because we have recently found that the level of edelfosine in plasma, after daily oral administration of 30 mg/kg, was about 10.3–25.2 µM in both BALB/c and immunodeficient mice [29], [30], [49], a dose that was effective in inhibiting cancer cell growth *in vivo* [29], [30], [50], our results indicate that edelfosine was active against all *Leishmania spp.* tested at pharmacologically relevant concentrations (Table 1).

### Edelfosine is the most potent ALP in inducing apoptosis-like cell death in *Leishmania* promastigotes

**Figure 2:**
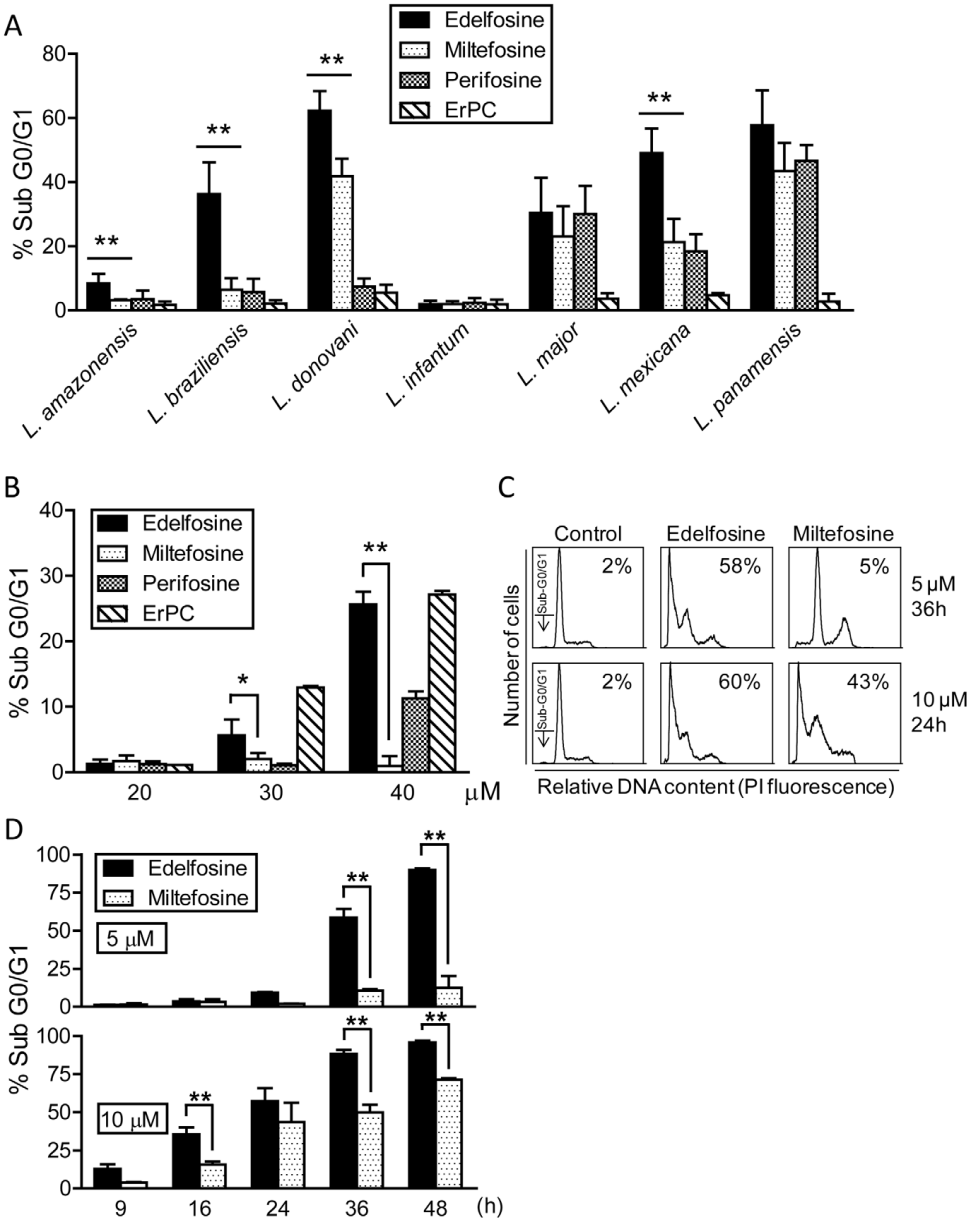
Differential ability of ALPs to induce apoptosis-like cell death in *Leishmania spp.* (A) Promastigotes from different *Leishmania spp.* were treated with 10 µM edelfosine, miltefosine, perifosine or erucylphosphocholine (ErPC) at 26°C for 24 h. Apoptosis-like cell death was then quantitated as percentage of parasites in the sub-G_0_/G_1_ region by flow cytometry. (B) *L. infantum* promastigotes were incubated with different concentrations of edelfosine, miltefosine, perifosine and erucylphosphocholine (ErPC) at 26°C for 24 h, and then apoptosis-like cell death was determined by flow cytometry. (C) Representative histograms of cell cycle analysis of *L. panamensis* promastigotes treated with 5 and 10 µM edelfosine and miltefosine at different incubation times. The position of the sub-G_0_/G_1_ peak, integrated by parasites undergoing apoptosis-like cell death, is indicated by arrows. Percentages of apoptotic parasites are shown in each histogram. (D) *L. panamensis* promastigotes were treated with 5 and 10 µM edelfosine or miltefosine at different incubation times, and then apoptosis-like cell death was determined by flow cytometry. Untreated *Leishmania* promastigotes were run in parallel, and apoptosis-like cell death was less than 1.5% in untreated parasites in all cases shown in panels A–D. Data are means ± SD or representative of four independent experiments. Asterisks indicate that the differences between edelfosine- and miltefosine-treated cells are statistically significant. (*) *P*<0.05. (**) *P*<0.01.

The above results showed that ALPs were able to inhibit *Leishmania spp.* proliferation at distinct rates. We next analyzed whether these agents, used at the pharmacologically relevant concentration of 10 µM, were able to induce an apoptotic-like cell death in *Leishmania spp.* promastigotes by determining DNA fragmentation by flow cytometry. Parasites displaying a sub-G_0_/G_1_ hypodiploid DNA content represent cells that undergo DNA breakdown and an apoptotic-like cell death [51]. We found that edelfosine was the most active ALP in promoting a potent apoptotic-like response in all *Leishmania spp.* tested (Figure 2A). The well nigh absence of apoptotic response in *L. infantum* promastigotes (Figure 2A) was expected, as ALPs were used at 10 µM, below the IC_50_ value for the inhibition of *L. infantum* promastigote proliferation measured by XTT assays (Table 1). Interestingly, edelfosine showed a much higher proapoptotic-like activity against *L. donovani* and *L. mexicana* promastigotes than miltefosine and perifosine (Figure 2A), despite the similar IC_50_ values (∼2–3 µM) of the three ALPs, assessed by XTT assays (Table 1). These results suggest that the induction of cell death by edelfosine might differ somewhat from the way by which miltefosine and perifosine promote parasite killing. The ability of the distinct ALPs to induce apoptosis-like cell death in *Leishmania spp.* promastigotes ranked edelfosine>perifosine≅miltefosine>erucylphosphocholine. Results shown in Figure 2A also show that the ability of edelfosine to promote an apoptosis-like cell death is highly dependent on the *Leishmania* sub-genus. In this regard, edelfosine inhibited proliferation of *L. amazonensis* (sug-genus *Leishmania*) and *L. braziliensis* (sug-genus *Viannia*) promastigotes with XTT IC_50_ values of 6.4 and 18.3 µM, respectively (Table 1), but the percentage of parasites with a sub-G_0_/G_1_ hypodiploid DNA content was higher in *L. braziliensis* than in *L. amazonensis* promastigotes (Figure 2A).

*L. infantum* promastigotes behaved somewhat different from other *Leishmania* species, with regard to their sensitivity to undergo apoptosis-like cell death by ALPs, requiring high ALP concentrations. A dose-response analysis of the apoptotic-like response of *L. infantum* promastigotes to the four ALPs tested was in agreement with the above XTT IC_50_ values of the corresponding drugs (cf. Figure 2B and Table 1), with erucylphosphocholine as the most efficient ALP at 30 µM (Figure 2B). However, at higher concentrations, edelfosine became as efficient as erucylphosphocholine in prompting an apoptotosis-like cell death in *L. infantum* promastigotes (Figure 2B).

A comparative dose-response analysis showed that edelfosine was more potent than miltefosine in inducing apoptosis-like cell death in *L. panamensis* promastigotes (Figure 2, C and D), edelfosine being highly effective even at 5 µM. These results agree with our above data on XTT IC_50_ figures (Table 1). The cell cycle profiles from propidium iodide-stained *L. panamensis* promastigotes showed a high percentage of parasites with apoptosis-like DNA breakdown following edelfosine treatment at either 5 or 10 µM (Figure 2, C and D), whereas miltefosine induced only a significant DNA breakdown response at 10 µM (Figure 2, C and D). Interestingly, edelfosine also induced apoptosis-like cell death in *L. panamensis* axenic amastigotes (25.8±4.6 and 55.4±2.8% sub-G_0_/G_1_ cells (*n* = 3) after 24 h incubation with 10 and 20 µM edelfosine, respectively).

### Edelfosine accumulates in intracellular *Leishmania* parasites

**Figure 3:**
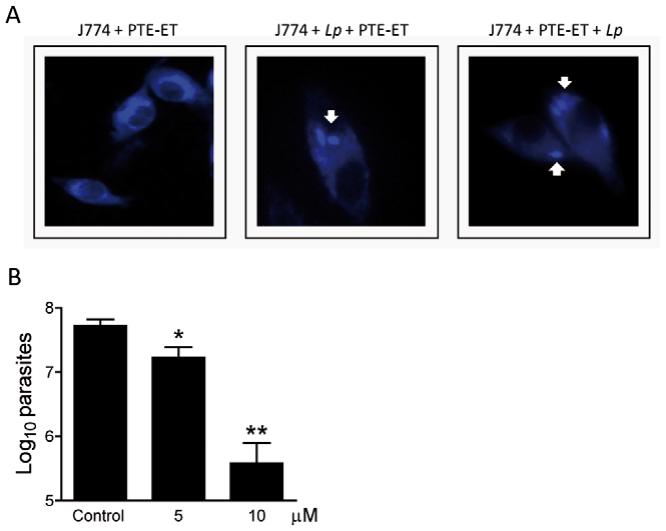
Antileishmanial activity of edelfosine against intracellular *Leishmania* amastigotes within macrophage-like J774 cells. (A) J774 cells, incubated with the blue-emitting fluorescent analog PTE-ET (*left panel*), or with *L. panamensis* (*Lp*) and then with PTE-ET (*middle panel*), or with PTE-ET and then with *L. panamensis* (*Lp*) (right panel), were analyzed by fluorescence microscopy to examine drug localization. White arrows point to the intracellular amastigotes. (B) Parasite burden in *L. panamensis*-infected J774 cells untreated (Control) and treated with 5 or 10 µM edelfosine for 24 h. Data are means ± SD or representative of four independent experiments. Asterisks indicate that the differences between control and edelfosine-treated groups are statistically significant. (*) *P*<0.05. (**) *P*<0.01.

Because *Leishmania* parasites use macrophages as their main host cell in mammalian infection, we next analyzed the localization of edelfosine in *Leishmania*-infected macrophages. To this aim, we used the fluorescent edelfosine analog *all*-(*E*)-1-*O*-(15′-phenylpentadeca-8′,10′,12′,14′-tetraenyl)-2-*O*-methyl-*rac*-glycero-3-phosphocholine (PTE-ET), which has been previously used as a *bona fide* compound to analyze the subcellular localization of edelfosine in cancer cells [30], [34], [46], [52], [53], and it fully mimics the antitumor [30], [34], [46], [52], [53] and antileishmanial [54] (data not shown) actions of the parent drug edelfosine. The mouse macrophage-like cell line J774 was rather resistant to undergo apoptosis following treatment with edelfosine (IC_50_ = 40.7±7.1 µM, assessed by XTT assays), and therefore it was used as a host cell line for *Leishmania* infection. Edelfosine (10 µM) was unable to induce apoptosis in J774 cells following 24 h incubation (<2% apoptosis), and caused less than 15% apoptosis after 48 h incubation. This is in stark contrast to the high sensitivity of other monocyte-like cell lines to edelfosine, such as human U937 cells [28], [55], [56], which undergo rapid apoptosis and can therefore not be used as host cells to analyze the effect of ALPs on intracellular parasites residing in macrophages. Incubation of J774 macrophages with PTE-ET showed that the fluorescent edelfosine analog was taken up into the cell (Figure 3A). The blue fluorescence of PTE-ET was mainly located around the nucleus (Figure 3A, left panel) that could be related to a predominant accumulation of this ether lipid in the endoplasmic reticulum of J774 cells, as previously reported for solid tumor cells [50], [52]. When macrophages were infected with *L. panamensis* parasites, an intense blue fluorescence was detected in the intracellular parasites (Figure 3A, middle panel), indicating that a major location of the PTE-ET fluorescent compound turned out to be in the intracellular parasites inside the macrophage. The PTE-ET location in the parasites residing in the macrophage was clearly detected, irrespective of whether PTE-ET was incubated with macrophages previously infected with parasites (Figure 3A, middle panel), or with intact macrophages and then subsequently incubated with parasites (Figure 3A, right panel). Macrophages containing a low number of *Leishmania* amastigotes are shown in Figure 3 in order to facilitate visualization of the fluorescent drug location in the parasites (Figure 3A). Similar data were obtained with primary mouse bone marrow-derived macrophages, which were resistant to 10 µM edelfosine, following infection with *L. major* (data not shown). These data suggest that edelfosine accumulates in intracellular *Leishmania* parasites inside macrophages, in a similar way as PTE-ET, to exert its anti-parasite action regardless drug treatment is before or after infection.

### Edelfosine induces cell death of *Leishmania* amastigotes inside macrophages

We also found that edelfosine efficiently killed J774 macrophage-residing *L. panamensis* by examining the parasitic burden of macrophages through limiting dilution assays (Figure 3B). The cytotoxic action of edelfosine against intracellular *L. panamensis* amastigotes was further confirmed by a dramatic decrease in the number of intracellular parasites, using J774 macrophages infected with green fluorescent *L. panamensis*, previously transfected with p.6.5-egfp to express green fluorescent protein (GFP) [57] (data not shown).

Some anti-parasite drugs are suggested to promote their action through the generation of nitric oxide (NO) [58], as this molecule exerts an important anti-parasitic effect [59], [60]. Miltefosine has been reported to induce NO in U937 cells [61]. However, we were unable to detect NO production following incubation of 10 µM edelfosine with J774 macrophages (<2 nmol nitrites/10^6^ J774 cells after 18 h incubation), unlike cell incubation with 10 µg/ml LPS (100 nmol nitrites/10^6^ J774 cells after 18 h incubation). Likewise, edelfosine treatment failed to prompt NO synthesis in mouse bone marrow-derived macrophages and rat alveolar macrophages (data not shown). These data suggest that the killing effect of edelfosine on macrophage-residing *Leishmania* parasites does not depend on NO generation.

### *In vivo* antileishmanial activity of edelfosine in a mouse model

**Figure 4:**
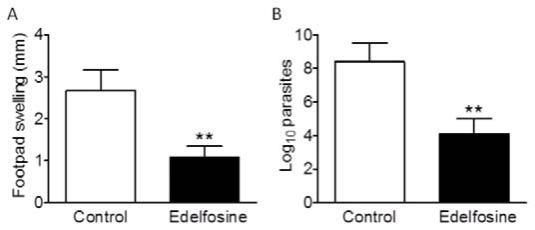
*In vivo* antileishmanial action of edelfosine in *L. major*-infected mice. BALB/c mice were infected with 2×10^6^ *L. major* promastigotes in the left hind footpad, and after swelling was perceptible, mice were randomized into drug-treated (15 mg edelfosine/kg of body weight, daily oral administration for 28 days) and control (water vehicle) groups of 7 mice each. After completion of the 4-week treatment, lesions were evaluated by measuring the footpad swelling (A) and determining the parasite load (B), using caliper measurements and limiting dilution assays respectively. Data are means ± SD (*n* = 7). Asterisks indicate that the differences between control and edelfosine-treated groups are statistically significant. (**) *P*<0.01.

We next examined the *in vivo* antileishmanial activity of edelfosine in BALB/c mice infected subcutaneously in the footpad with 2×10^6^ infective stationary-phase *L. major* promastigotes. In agreement with previous estimates [29], [30], [49], we found that a daily oral administration of 15 or 30 mg/kg edelfosine was well tolerated, 45 mg/kg being the maximum tolerated dose, following toxicity analyses, where animals were monitored for body weight loss or any appreciable side-effect, including changes in strength and general condition (data not shown). We found that a daily oral administration dose of 15 mg/kg body weight edelfosine achieved a remarkable inhibition of both footpad inflammation (Figure 4A) and parasitic load (Figure 4B), as assessed by caliper measures of footpad swelling and limiting dilution assays, respectively, at the end of the 28-day treatment period. In comparison experiments, we found that oral treatment of *L. major*-infected BALB/c mice with edelfosine was slightly more effective than with miltefosine, although differences were not statistically significant (data not shown). The dose of edelfosine used in our assays was similar to the dose used for miltefosine in mouse models, ranging from 2.5 to 25 mg/kg of body weight/day given orally, and being 20 mg/kg/day the most widely used dose for *in vivo* murine experiments [35], [39], [62]–[65]. In addition, because the molecular masses for edelfosine and miltefosine are 523.7 and 407.6, respectively, the edelfosine dose used in our assays (15 mg/kg, corresponding to 28.6 µmol/kg) was even lower than the usual miltefosine dose (20 mg/kg, corresponding to 49.1 µmol/kg) in these *in vivo* murine studies.

### *In vivo* antileishmanial activity of edelfosine in hamster models of cutaneous and mucocutaneous *leishmaniasis*

**Figure 5:**
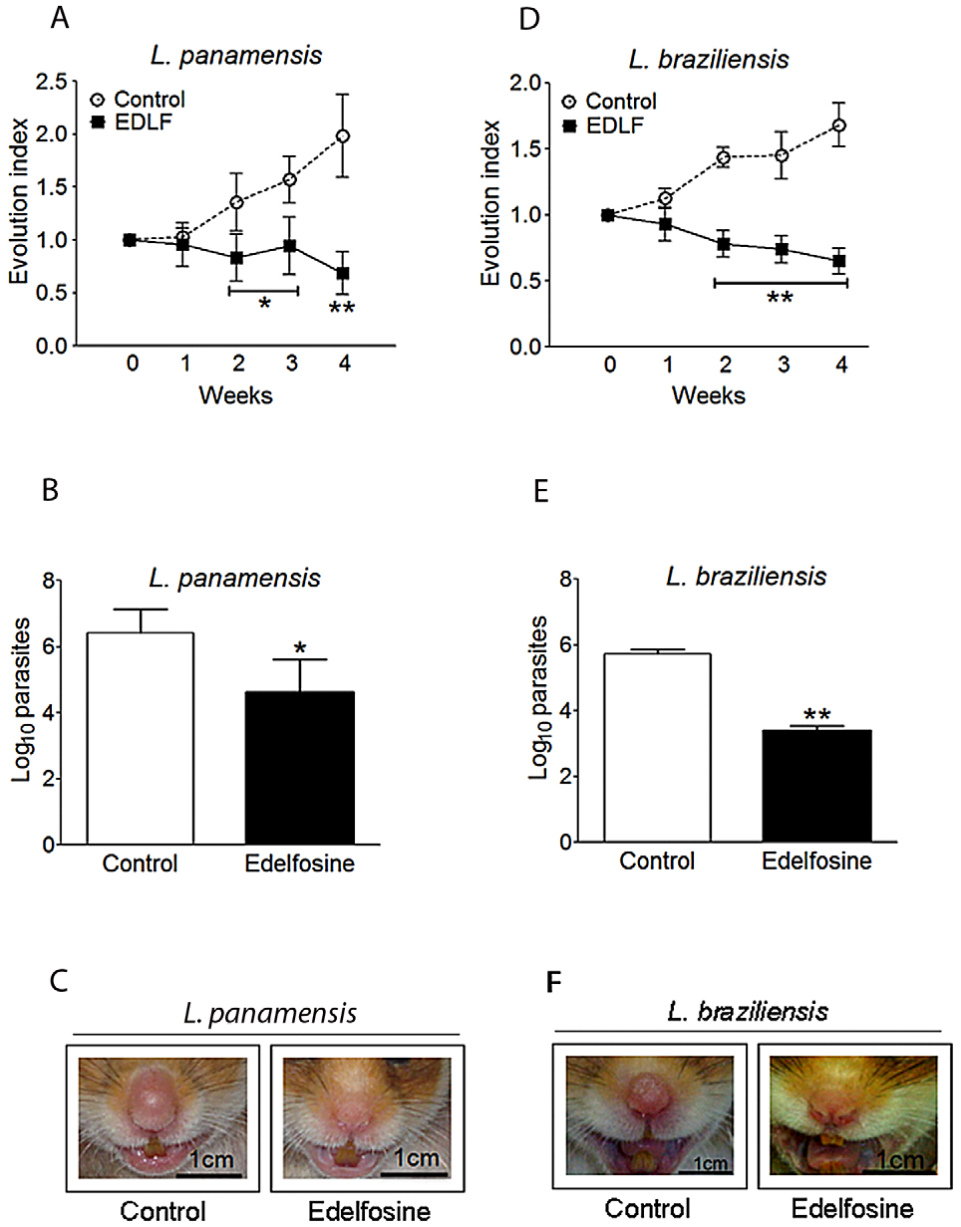
*In vivo* antileishmanial action of edelfosine in *L. panamensis- and L. braziliensis*-infected hamsters. Golden hamsters were infected with 1×10^6^ *L. panamensis* or *L. braziliensis* promastigotes in the nose, and after swelling was perceptible, hamsters were randomized into drug-treated (26 mg edelfosine/kg of body weight, daily oral administration for 28 days) and control (water vehicle) groups of 7 hamsters each. (A, D) Lesion development was monitored by measuring nose thickness at regular intervals, and comparison to values obtained before treatment (evolution index). (B, E) Parasite load was determined by limiting dilution assays after completion of the 4-week *in vivo* assays. Data are means ± SD (*n* = 7). Asterisks indicate that the differences between control and edelfosine-treated groups are statistically significant. (*) *P*<0.05. (**) *P*<0.01. (C, F) Edelfosine treatment led to a dramatic decrease and amelioration in parasite-induced nose thickness and damage, as shown by representative photographs from drug-free control and edelfosine-treated hamsters.

Next, we used golden hamsters as an additional experimental animal model of leishmaniasis. Hamsters have been reported to better reproduce the clinicopathological features of human leishmaniasis than mice [66]–[68]. One million promastigotes of *L. panamensis* and *L. braziliensis* were inoculated in the nose of golden hamsters, as animal models for cutaneous and mucocutaneous leishmaniasis, since the above *Leishmania* species can cause both cutaneous and mucocutaneous disease [69], [70]. Then, hamsters were randomized into drug-treated and drug-free control (water vehicle) groups of seven hamsters each, and the animal models for *L. panamensis* (Figure 5, A–C) and *L. braziliensis* (Figure 5, D–F) infections were monitored for the antileishmanial efficacy of edelfosine. Serial caliper measurements during the course of the assays were made to determine the rate of nose swelling (Figure 5, A and D). Progression of the disease led to a dramatic swelling and ulceration of the nose. Oral administration of edelfosine (26 mg/kg body weight) on a daily basis for 4 weeks (28 days) induced a remarkable decrease in both nasal swelling and parasitic load at the site of infection (Figure 5). This dose is lower than the miltefosine dose (40 mg/kg/day) used in a recent study with *L. donovani*-infected hamsters [71]. Here, we found no appreciable adverse effects on the general condition of the animals following a daily oral administration of 26 mg/kg edelfosine. The effect of edelfosine on nose swelling became evident in both *L. panamensis* and *L. braziliensis* infections after two weeks of treatment (Figure 5, A and D). The parasite loads, assessed by limiting dilution assays, were significantly diminished in both animal models following oral treatment with edelfosine (Figure 5, B and E). Untreated infected animals displayed intense swelling and ulceration in their noses, but edelfosine treatment greatly ameliorated the signs of leishmaniasis (Figure 5, E and F).

### Edelfosine shows potent *in vitro* and *in vivo* antileishmanial activity against SbV-resistant *L. panamensis* parasites

**Figure 6:**
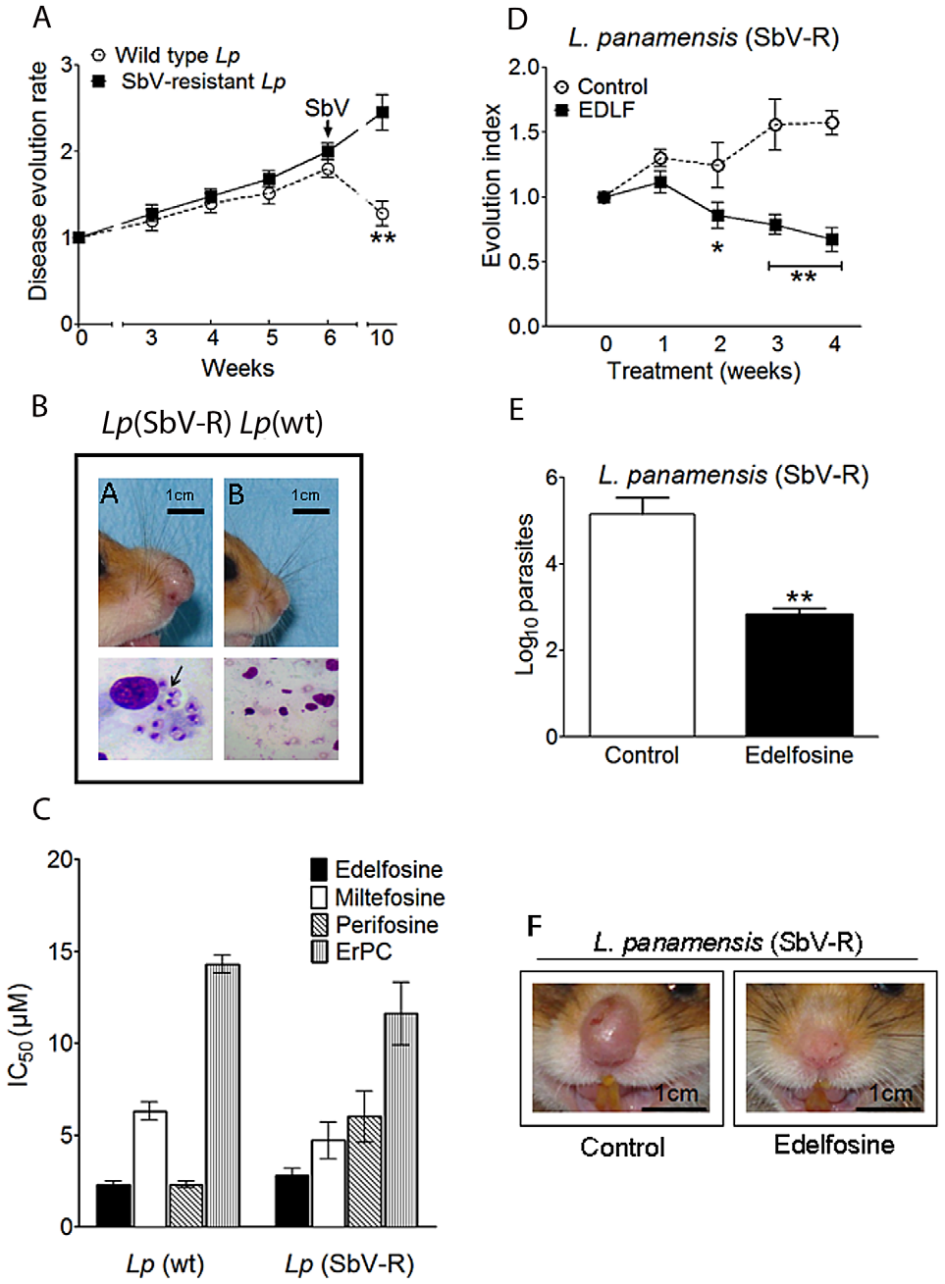
Sensitivity of SbV-resistant *L. panamensis* parasites to edelfosine. (A) Two groups of eight golden hamsters each were infected in the nose with wild type and SbV-resistant *L. panamensis* (*Lp*) promastigotes, and after the sixth week post-infection, they were treated with a daily intramuscular injection of Glucantime (SbV) for 4 weeks. Disease evolution rate was measured along the whole process through determining nose thickness as compared to figures obtained before infection. (B) Golden hamsters inoculated with SbV-resistant (SbV-R) *L. panamensis* (*Lp*) did not respond to treatment with Glucantime (inflamed nose) (upper left panel), and nose smears showed amastigotes within macrophages (arrow) following Giemsa staining (lower left panel). However, hamsters infected with wild type (wt) *L. panamensis* (*Lp*) fully responded to Glucantime treatment, showing uninflamed nose and negative staining for amastigotes in nose smears (upper and lower right panels). (C) IC_50_ values of edelfosine, miltefosine, perifosine, and erucylphosphocholine (ErPC) for *in vitro* growth inhibition of wild type (wt) and SbV-resistant (SbV-R) *L. panamensis* (*Lp*) promastigotes were determined by XTT assays. (D–F) Golden hamsters were infected with 1×10^6^ SbV-resistant *L. panamensis* promastigotes in the nose, and after nose inflammation was evident, hamsters were randomized into drug-treated (26 mg edelfosine/kg of body weight, daily oral administration for 28 days) and control (water vehicle) groups of 7 hamsters each. (D) Lesion development was monitored by measuring nose thickness at regular intervals and comparison to values obtained before treatment (evolution index). (E) Parasite load was determined by limiting dilution assays after completion of the 4-week *in vivo* assay. (F) Edelfosine treatment led to a dramatic decrease and amelioration in parasite-induced nose thickness and damage, as shown by photographs from drug-free control and edelfosine-treated hamsters. Data are means ± SD or representative experiments (*n* = 7). Asterisks indicate that differences between control and edelfosine-treated groups, or between wild type and SbV-resistant parasites treated with SbV, are statistically significant. (*) *P*<0.05. (**) P<0.01.

Cutaneous leishmaniasis is the most common form of leishmaniasis and is endemic in many tropical and subtropical countries [1], [2]. Common therapies for leishmaniasis for more than 60 years include the use of SbV drugs as meglumine antimoniate (Glucantime) or sodium stibogluconate (Pentostam) [2], [72]. However, extensive use of these compounds is leading to SbV resistance. Thus, parasites have become resistant to antimony in many parts of the world, and primary resistance to SbV exceeds 60% of cases of leishmaniasis in the state of Bihar in India [73]. Different *Leishmania* species have been shown to display distinct susceptibility to antimonials [74], [75]. In addition, susceptibility of *L. donovani* to SbV has been reported to follow stage transformation from promastigotes to axenic amastigotes, while resistance to SbV is acquired when amastigotes differentiate into promastigotes [76]. SbV has also been reported to be active, even though to different degrees, against a number of *Leishmania spp.* promastigotes and amastigotes *in vitro*, including *L. panamensis* [77]–[81]. On these grounds and because of possible clinical implications, we generated a SbV-resistant *L. panamensis* strain to be tested for the antiparasitic activity of the distinct ALPs. Induction of resistance to SbV in *L. panamensis* promastigotes was achieved by continuous *in vitro* exposure of these parasites to increasing Glucantime concentrations for 1 year. The SbV-resistant *L. panamensis* strain was able to resist concentrations of Glucantime as high as 36 mg/ml, as assessed by XTT assays, a concentration 9-fold higher than the IC_50_ (4 mg/ml) for wild type *L. panamensis* promastigotes. Because of the different susceptibility to SbV shown by certain *Leishmania spp.*, depending on their promastigote or amastigote stage, SbV resistance of *L. panamensis* promastigotes was further evaluated by *in vivo* experiments in a hamster model. Two groups of eight hamsters each were inoculated in the nose with wild type and SbV-resistant *L. panamensis* promastigotes, and then, after a 6-week post-infection period, when nose swelling was clearly detected, hamsters were injected intramuscularly with 40 mg/kg body weight Glucantime (SbV), on a daily basis for 4 weeks. As shown in Figure 6 (A and B), swelling was decreased in animals infected with wild type *L. panamensis*, but increased in animals infected with SbV-resistant *L. panamensis*. In addition, macrophages infected with *Leishmania* amastigotes were readily observed in smears from the nose of SbV-resistant *L. panamensis*-infected hamsters, but not from wild type *L. panamensis*-infected animals, treated with SbV (Figure 6B). Moreover, the parasitic burden in the nose of the two groups of animals indicated that the amount of viable wild type *L. panamensis* was dramatically diminished following treatment with the pentavalent antimonial drug, but the SbV-resistant *L. panamensis* parasites remained viable in the *in vivo* assay (data not shown). These results indicate that the generated SbV-resistant *L. panamensis* strain was highly resistant to pentavalent antimonial treatment both *in vitro* and *in vivo*.

Next, we tested *in vitro* the activity of the four ALPs edelfosine, miltefosine, perifosine and erucylphosphocholine against both wild type and SbV-resistant *L. panamensis* promastigotes by XTT assays. We found that all ALPs were effective in inhibiting proliferation of SbV-resistant *L. panamensis* promastigotes showing similar IC_50_ values to those found against wild type *L. panamensis* (Figure 6C). Edelfosine was the most effective ALP against SbV-resistant *L. panamensis* promastigotes and no difference in edelfosine sensitivity was observed between wild type and SbV-resistant strains (Figure 6C).

Infection of hamsters with SbV-resistant *L. panamensis* parasites in the nose, showed that a daily oral treatment with edelfosine (26 mg/kg body weight) for 4 weeks led to a dramatic decrease in the evolution index, parasitic burden and local inflammation (Figure 6, D–F). The first signs of improvement were detected after about two weeks of treatment (Figure 6A). These data indicate that oral treatment with edelfosine was efficient against leishmaniasis caused by SbV-resistant *L. panamensis* parasites.

### Differential time requirement for the generation of resistance to edelfosine and miltefosine in *Leishmania spp.* promastigotes

A major concern in anti-parasitic chemotherapy is the generation of drug resistance. Thus, we next analyzed the feasibility to generate drug resistance to miltefosine and edelfosine in different *Leishmania* species, by a gradual increase in drug concentration. We determined the time required to achieve resistance to 30 µM miltefosine or edelfosine. This drug concentration could be appropriate to distinguish between specific and unspecific effects, and thereby drug resistance was considered when parasites became resistant to a final drug concentration of 30 µM. We found that the continuous exposure of *L. donovani*, *L. major* and *L. panamensis* promastigotes to increasing amounts of miltefosine led to a rather rapid advent of drug resistance following 40–64 days of treatment (Table 2). However, relatively more protracted continuous treatments were required to generate edelfosine resistance in *L. major* and *L. panamensis* promastigotes (Table 2). Interestingly, whereas miltefosine treatment led to drug resistance in *L. donovani* after a relatively short period of time (Table 2), no drug resistance was detected after 100-day treatment of *L. donovani* with edelfosine (Table 2).

**Table 2:** Differential incubation times required for drug resistance generation.

*Leishmania* species	Time required for drug resistance (days)	
Promastigotes	Edelfosine	Miltefosine
*L. donovani*	NR	40
*L. major*	88	60
*L. panamensis*	89	64

*Leishmania* promastigotes were incubated with increasing concentrations of edelfosine and miltefosine until parasite viability in the presence of 30 µM drug was over 80% (considered as drug resistant). The maximum period of time evaluated for acquisition of the resistant phenotype was 100 days, and no resistance to edelfosine was generated in *L. donovani* promastigotes after this incubation time. NR, no resistance.

## Discussion

Our results show the *in vitro* and *in vivo* antileishmanial activity of edelfosine against different *Leishmania* species. The ability of edelfosine to kill distinct *Leishmania spp.* promastigotes and amastigotes is in general higher than other ALPs, and the antileishmanial activity of ALPs ranked edelfosine>perifosine>miltefosine>erucylphosphocholine. Edelfosine also shows a higher capacity to induce an apoptosis-like cell death in *Leishmania* than miltefosine (Impavido), which has been approved as the first oral drug active against visceral leishmaniasis [2]. However, recent studies have challenged the efficacy of miltefosine against some cutaneous leishmaniasis [13]–[15], [17]–[20], and relapse cases of miltefosine-treated parasites have been reported in visceral and diffuse cutaneous leishmaniasis [82]–[84] as well as in HIV-positive patients [85], [86].

Here, we have found that edelfosine shows an outstanding activity against a wide number of *Leishmania spp.* causing cutaneous, mucocutaneous and visceral leishmaniasis. Edelfosine was able to kill parasites in both promastigote and amastigote forms through an apoptosis-like process that involved DNA degradation, as assessed by an increase in the percentage of cells with a hypodiploid DNA content. *Leishmania* parasites infect macrophages wherein they reside and replicate in a fusion competent vacuole (parasitophorous vacuole). Interestingly, edelfosine efficiently killed intracellular parasite amastigotes inside macrophages, without affecting the host cells. This killing activity on intracellular parasites seems to be mainly due to a direct action of the drug on the parasite, as edelfosine was unable to induce NO generation in macrophages, while a fluorescent edelfosine analog accumulated in the intracellular parasites within macrophages.

Our data also reveal a remarkable antileishmanial activity of edelfosine in several *in vivo* assays using mouse and hamster animal models infected with *L. major*, *L. panamensis* or *L. braziliensis*. To our knowledge this is the first study using hamsters as animal models for the *in vivo* evaluation of ALPs against cutaneous leishmaniasis. In addition, both *in vitro* and *in vivo* evidence showed that edelfosine was very effective against SbV-resistant *Leishmania* parasites. This is of importance as pentavalent antimonials Glucantime and Pentostam are being used in the treatment of leishmaniasis for over more than six decades, and still they are the first line drugs of choice and the traditional treatment worldwide. However, resistance to pentavalent antimonials is emerging as a result of their widespread use. A stark example of SbV resistance is well documented in Bihar (India), which houses approximately 90% of Indias's cases of visceral leishmaniasis, representing about 50% of the world's cases, and where resistance ended the usefulness of SbV more than a decade ago [2].

A major potential drawback in the use of miltefosine could be the relatively rapid generation of drug resistance *in vitro*. We have found here that generation of drug resistance required longer incubation times of *Leishmania spp.* with edelfosine than with miltefosine. Furthermore, whereas miltefosine generated drug resistance in *L. donovani* following a 40-day treatment, no resistance to edelfosine was detected after 100-day incubation.

It is worthwhile to note that miltefosine treatment has been reported to be unsatisfactory against infections caused by *L. braziliensis* [13]–[15], [17]–[20], whereas here we have found a remarkable antiparasitic activity of edelfosine in *L. braziliensis*-infected hamsters. In addition, edelfosine offers a number of additional advantages as compared to miltefosine, such as the fact that edelfosine shows a potent anti-inflammatory action [87], and no apparent toxicity [87]. *Leishmania* parasites enter first neutrophils through the regulation of granule fusion processes that prevents any deleterious action on the parasite [88]. *Leishmania* parasites use polymorphonuclear neutrophils as intermediate hosts before their ultimate delivery to macrophages, following engulfment of parasite-infected neutrophils, and in this way *Leishmania* can escape the host immune system [89]. A significant part of the destruction caused by cutaneous leishmaniasis is due to severe inflammation at the site of infection in the skin, leading to ulceration [90]. Neutrophils are recruited into the site of infection during cutaneous leishmaniasis [91], [92], and accumulation of neutrophils have been linked to tissue damage [93]. Edelfosine induces L-selectin (CD62L) shedding, and thus prevents neutrophil extravasation to the inflammation or infection site [87]. On these grounds, leishmaniasis could be ameliorated by oral treatment of edelfosine, which could reduce the parasite burden, by direct parasite killing, as well as the ulcerative process and subsequent scar formation, by a reduction in the recruitment of neutrophils into the site of infection.

A serious drawback of miltefosine is its teratogenic effects [24], however no studies have been conducted so far for a putative teratogenic action of edelfosine.

The studies reported here provide compelling evidence for the potent antileishmanial activity of edelfosine, which together with the low toxicity profile displayed by this ether lipid and its anti-inflammatory activity, warrants further clinical evaluation as a possible alternative treatment against leishmaniasis.
